# Numerical Simulation and Experimental Investigation on Two Strengthening Schemes of Cantilever Brackets

**DOI:** 10.3390/ma15217655

**Published:** 2022-10-31

**Authors:** Guilin Sheng, Guangyuan Li, Liming Zhu, Zhiyong Zhou, Wenfeng Du

**Affiliations:** 1 Institute of Steel and Spatial Structures, College of Civil Engineering and Architecture, Henan University, Kaifeng 475004, China; 2 Railway Municipal Environmental Construction Co., Ltd., Shanghai 200000, China; 3 Department of Engineering Mechanics, Northwestern Polytechnical University, Xi’an 710072, China

**Keywords:** cantilever bracket, numerical simulation, experimental investigation, bearing capacity

## Abstract

Cantilever brackets have been widely used in buildings to provide support for all kinds of pipes. In order to improve the bearing capacity of cantilever brackets, two types of reinforcement schemes are proposed, one is to sleeve a pipe, and the other is to add a haunch. Their mechanical properties are studied by numerical simulation and experimental investigation. First, the non-linear finite element (FE) simulation analysis was carried out, and the structural bearing capacity, stress distribution, and failure modes were discussed. Then, the full-scale model tests were completed to provide validation of the FE analysis. On this basis, a comparison of the FE results and test results of three kinds of cantilever brackets was discussed in detail. The results show that two reinforcement schemes can enhance the bearing capacity of the cantilever bracket significantly by 38.3% and 25.9%, respectively, and they are applicable for the reinforcement of existing cantilever brackets.

## 1. Introduction

As an important part of the support and hanger system, assembled cantilever brackets is widely used in all kinds of buildings, such as hotel, shopping mall, subway, and comprehensive pipe gallery [1,2,3,4]. As a non-structural component, assembled cantilever bracket is mainly used to connect building structures and electromechanical systems, and it is very important to give full play to the function of the building. The investigation shows that the investment of non-structural components in the investment of hospitals, office buildings, and hotels accounts for 92%, 87%, and 82%, respectively [5,6,7]. Therefore, the study of the comprehensive mechanical properties of the assembled cantilever bracket to prevent accidental disaster losses is of great importance.

In the early stage, there are few researches on the mechanical properties of assembled cantilever brackets; some problems, such as lack of corresponding design standards and over-reliance on designers’ experiences, are outstanding. In order to solve these problems, some scholars have begun to study the mechanical properties of cantilever brackets by experimental method or FE method in recent years. The model test research has an important safety guarantee for the practical engineering application of structures. The finite element simulation research has high accuracy, and it has a high consistency with the test results, which can provide some data reference [8,9,10]. Zuo [11] carried out the variable parameter analysis of aluminum alloy assembled cantilever brackets by using the FE program ABAQUS to study the failure mode and bearing capacity of specimens with different plate thicknesses, reinforcement inclination, tooth specifications, and different stress states. Li [12] investigated the bearing capacity of the composite cantilever bracket. By comparing the static FE simulation with the test results of the three-cantilever bracket, it was concluded that the three-cantilever bracket could meet the strength requirements under static action better. Zhong [13] proposed the calculation model of the bracket for electric power communication according to the force form of the bracket, which provides an effective reference for the calculation of the bearing capacity and displacement of the bracket. Li [14] carried out bearing capacity experiments on eight kinds of full-scale support models to study the influence of different geometric parameters on the bearing capacity displacement of the bracket. The results show that the bearing capacity of the bracket with stiffening form is the largest. Yuan [15] studied the bearing capacity and failure state of the new type of adjustable assembled cantilever bracket by model test and numerical simulation. From the research results, it can be concluded that the new type of assembled cantilever bracket can meet the requirements of the specification. Under the ultimate load, shear failure is easy to occur at the bolt hole. Cristopher [16] studied the effect of the opening ratio on the bearing capacity of single-leg cold-formed thin-walled steel. The test results show that the opening in the web reduces the bearing capacity of members, and if the size of the opening is large, brittle failure occurs. For an anti-seismic support hanger, it is necessary to consider the selection and design of an anti-seismic system and seismic bracing, tension bar, and connector.

In recent years, some scholars have studied earthquake damage and found that the fabricated pipeline system is easy to cause secondary earthquake damage, which brings difficulties to the repair after the earthquake [17,18]. Therefore, some scholars successively studied the seismic performance of the support and hanger. Zaghi [19] used a shaking table to conduct an experimental study on the pipeline system. Through the analysis of the experimental results, it can be concluded that the seismic fixed restraint has a good limiting effect on the displacement response of the support and hanger. Filatrault [20] adopted the new cyclic loading system to obtain the ultimate bearing capacity of the seismic hanger, which is used to determine the hysteretic performance of the hanger. Shang [21] studied the ultimate bearing capacity of three kinds of seismic support hangers through a quasi-static test and concluded that the screw-type seismic support hanger has the largest ultimate bearing capacity. On this basis, tests were carried out on the supports with different rod diameters. The results show that the rod diameter has little effect on the seismic capacity of the support, but the support with a small diameter has a greater deformation capacity. Wood [22] carried out the relevant experimental research on the anti-seismic hanger and obtained the load–displacement hysteresis curve. The test results show that the connector has a great influence on the seismic performance of the hanger.

With the rapid development of the communication industry, more and more communication cables are laid on the existing brackets, increasing the load of the original brackets. From the perspective of economy and safety, these loads are usually small, and it is most appropriate to take appropriate strengthening measures for the existing brackets. Most of the previous studies on cantilever support analyzed the mechanical properties of the members themselves or studied the effects of different parameters on the mechanical properties of the members, which are not suitable for solving the problems caused by adding small loads on the basis of the original cantilever members. This paper is mainly based on the idea of “reinforcement”, changing the structural form of the cantilever support, and studying the comprehensive mechanical properties of the cantilever support. Secondly, by comparing the mechanical properties of different structural forms of cantilever support, the appropriate type of cantilever support can be selected according to the actual needs of the project. Therefore, this work proposed two new types of reinforcement schemes on the basis of the original cantilever bracket, and the bearing capacity, stress distribution, and failure mode were simulated by the FE method based on ANSYS Workbench. Then, the quasi-static tests of three kinds of cantilever brackets were carried out by a self-balancing reaction frame, the test results were verified with the numerical simulation results, and the mechanical properties of the new cantilever brackets were comprehensively evaluated.

## 2. Specimen Design

**Figure 1:**
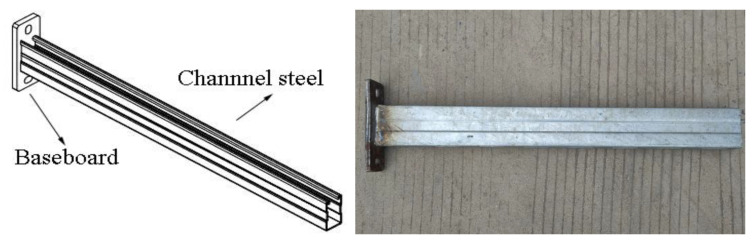
Model diagram of original cantilever bracket.

The original cantilever bracket is mainly composed of channel steel and baseboard. The channel steel and baseboard are connected by welds. This stereotyped product has been widely used in engineering projects. The model is shown in Figure 1.

**Figure 2:**
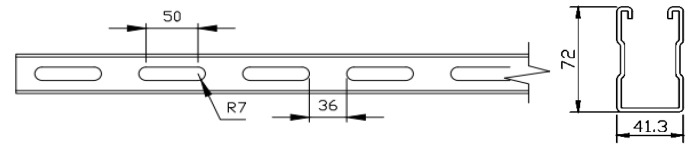
MC-72 channel steel.

**Figure 3:**
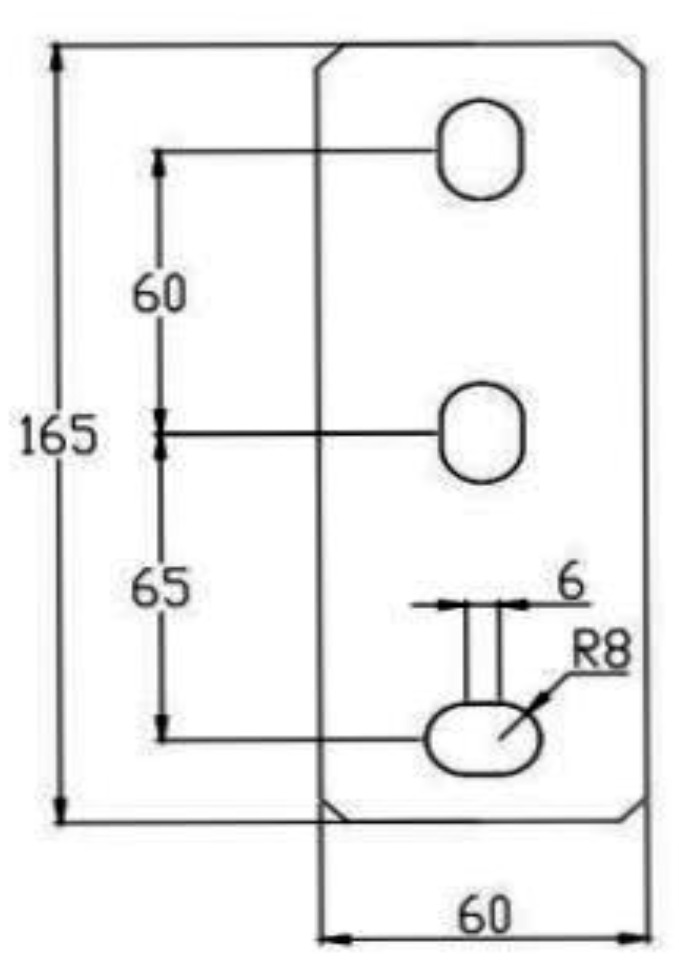
Baseboard.

Channel steel is the main bearing member of the cantilever bracket, with a design length of 800 mm, a section height of 72 mm, a width of 41.3 mm, a wall thickness of 2.75 mm, and a long round hole opened in the channel steel web; the side length is 50 mm, the circle radius is 7 mm, and the dimension details are shown in Figure 2. The specific size of the baseboard is 165 mm × 60 mm, with a thickness of 12 mm, as shown in Figure 3.

**Figure 4:**
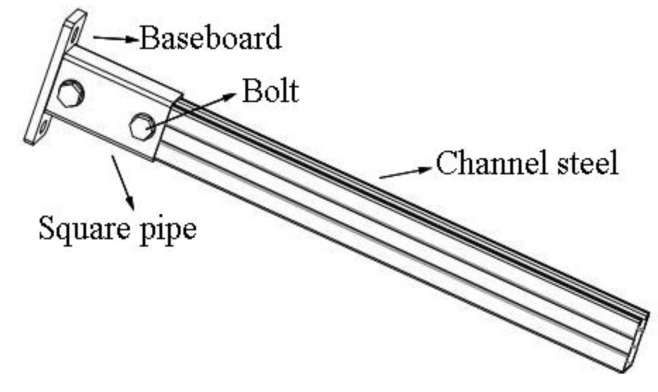
Model of S1.

**Figure 5:**
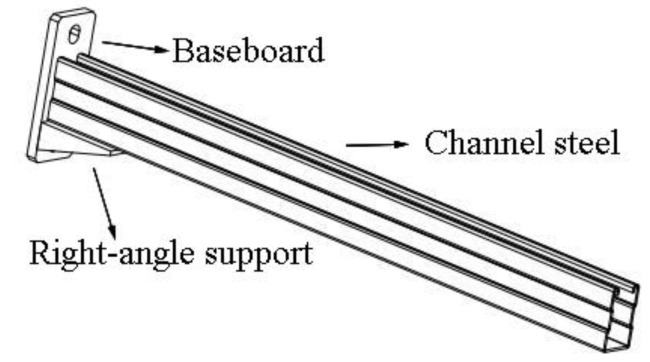
Model of S2.

Under the vertical load, the fixed end of the cantilever bracket bore the largest bending moment. Based on the principle of improving bending stiffness, this paper proposes two types of reinforcement schemes. The first (S1) is to sleeve a pipe, as shown in Figure 4. S1 is mainly composed of channel steel, baseboard, M16 bolts, and square sleeve pipe. The channel steel connects with the sleeve pipe by bolts. The sleeve pipe and the baseboard are welded, and the channel steel is in close contact with the baseboard. The size of the square sleeve pipe is 150 mm × 50 mm × 80 mm, and the wall thickness is 3 mm. The second (S2) is to add a haunch, as shown in Figure 5. S2 is mainly composed of channel steel, baseboard, and right-angle support. The channel steel connects with the baseboard by welding, and at the same time, the right-angle support is welded at the bottom of the channel steel to improve the overall stiffness and bearing capacity of the cantilever bracket. The right-angle support is made of one transverse plate, and two right-angle plates the size of the transverse plate is 100 mm × 60 mm × 6 mm, the size of the right-angle plate is 100 mm × 31 mm × 6 mm, and the angle between the oblique edge of the right-angle plate and the baseboard is 75°. The detailed dimensions of the components of the two types of cantilever brackets are shown in Table 1.

## 3. Numerical Simulation

### 3.1. Material Constitutive Model

The channel steel is S250GD steel, whose elastic modulus E is 210 GPa and Poisson’s ratio is 0.3. The other components of the cantilever brackets are Q235B steel, with the elastic modulus E 206 GPa and Poisson’s ratio of 0.3. The strengthening modulus E_T_ is 0.01E. The specific material properties are shown in Table 2.

**Figure 6:**
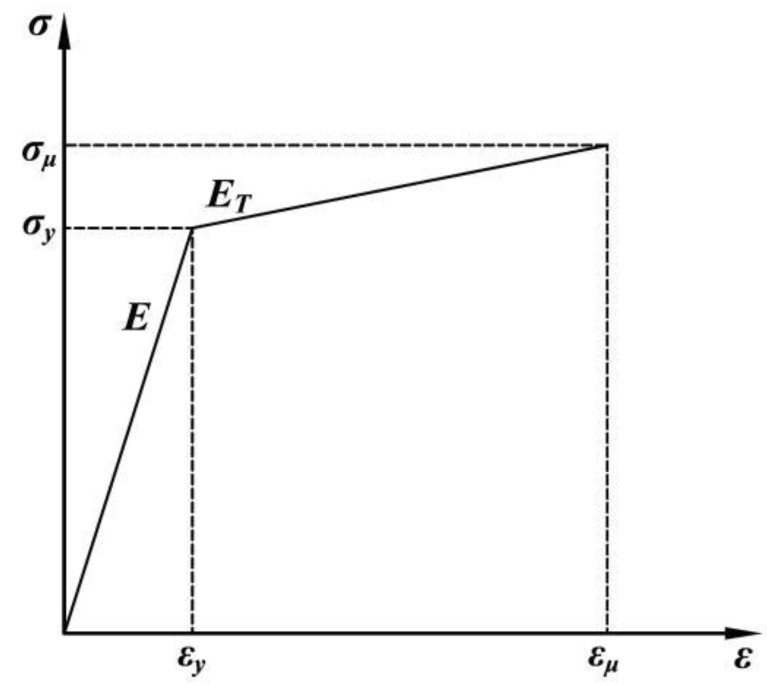
Constitutive relation of steel.

Material nonlinearity, large deformation, and contact nonlinearity were taken into account in the FE simulation. The development law of material plasticity and stiffness followed the Von Mises yield criterion. The stress–strain curves of steel adopted an ideal double-line isotropic strengthening model. The constitutive model is shown in Figure 6.

### 3.2. Analysis Model

The solid model was established by SolidWorks and imported into ANSYS Workbench for FE simulation. In the FE analysis, the model was properly simplified, and the initial geometric defect and the residual stress caused by welding were not considered [23,24]. For the components connected by welds, the binding contact was used. The model used hexahedral high-order solid element Soild186, which has a total of 20 nodes and can well simulate irregular geometric models. In the cantilever brackets with square tubes, the main function of bearing type bolt was to transfer the load borne by channel steel to square pipe, and the yield strength of the bolt was much higher than that of channel steel or square tube. In order to improve the calculation efficiency, the bolt was modeled simply, and the cylindrical model was adopted [25,26]. The contact nonlinearity was considered in the FE simulation, and the surface-to-surface contact was used to reflect the load transfer between members. The stiffness of each member of the cantilever bracket was similar, and the flexible–flexible contact form was adopted [27,28]. When setting the contact surface of the cantilever bracket, the channel steel was the contact surface, and the square tube and right-angle support were the target surface. In order to make the nonlinear problem converge more easily, the Augmented Lagrange contact algorithm was used to solve the problem, and the Gauss point method was used for contact detection. Binding contact was used between bolt and bolt hole, friction contact was used between square tube and channel steel, and the friction coefficient was 0.3.

### 3.3. Meshing and Boundary Setting

**Figure 7:**
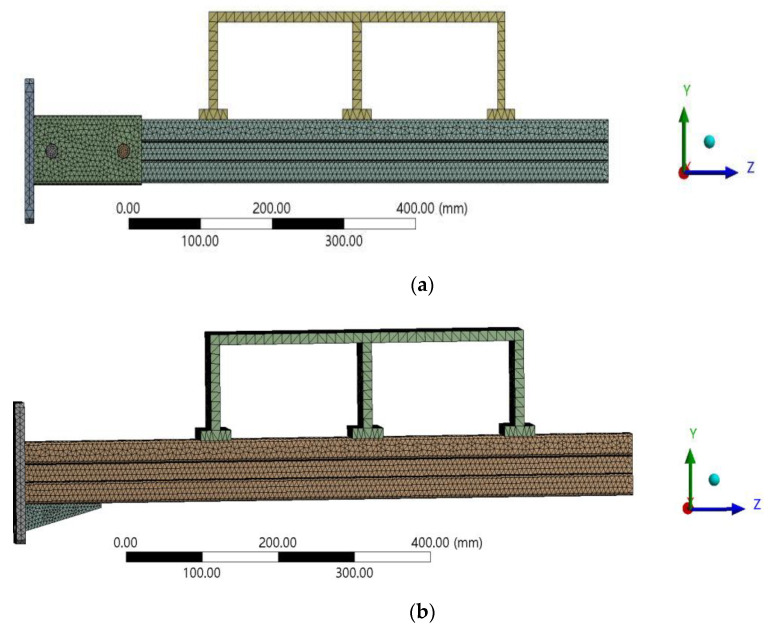
FE model: (**a**) S1, (**b**) S2.

The cantilever bracket model was irregular, which could be divided by the combination of the tetrahedral grid, hexahedral grid, and mapping division. The contact surface and the key components were refined, and the bolts were divided by mapping to obtain a better mesh. The mesh size was controlled at 3 mm, and the channel steel mesh size was controlled at 6 mm. The baseboard of the cantilever brackets adopted fixed constraints to restrict the displacement and rotation in the x, y, and z directions. In practical engineering, the pipeline is generally fixed to the channel steel by clamping. In order to truly simulate the stress state of the cantilever bracket, the loading beam was used to simulate the load caused by the pipeline to the cantilever bracket in the FE simulation. The load acts on the loading beam along the negative direction of the *y*-axis. After meshing, the structural models are shown in Figure 7a,b.

### 3.4. FE Simulation Results

Meshing is an important part of the pre-processing stage of FE, and the quality of the mesh directly affects the accuracy of FE results. In order to obtain the accurate solution of finite element simulation more efficiently, this paper refined the mesh of bolts and other key nodes or parts. In order to verify whether the FE results are reasonable, it is necessary to analyze the sensitivity of mesh size. For example, in the FE simulation of S1, keep the constraints and load values unchanged through the continuous refinement of the mesh, obtain different FE results, and observe whether the adjacent solutions are close. As can be seen from Table 3, the result errors of the three groups of data were all less than 5%, so as to verify the rationality of the results.

**Figure 8:**
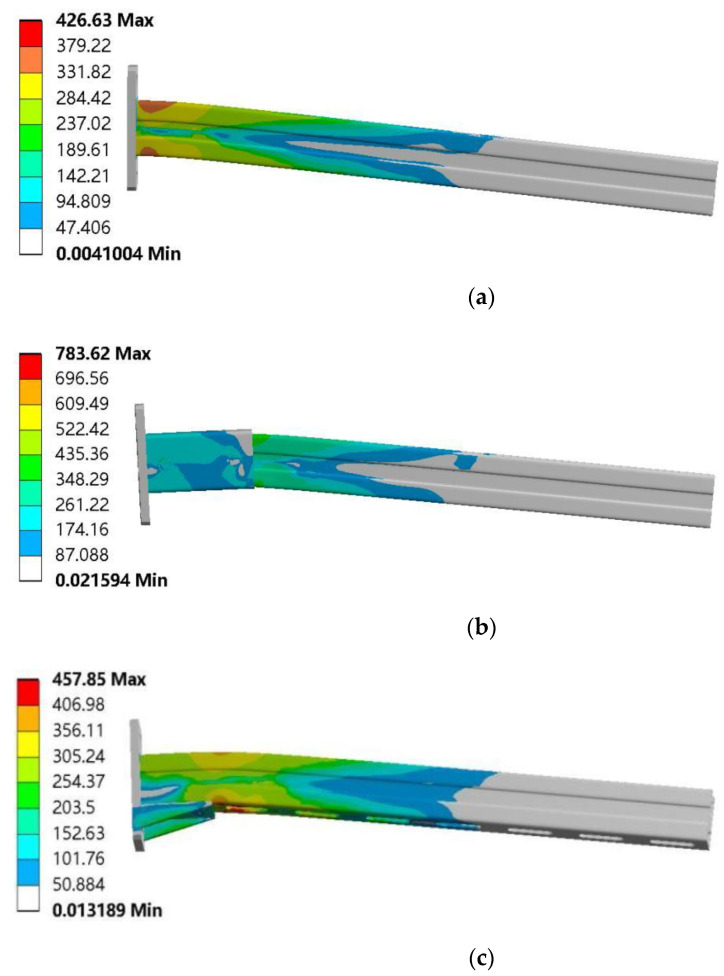
FE results: (**a**) original cantilever bracket, (**b**) S1, (**c**) S2.

The FE results are shown in Figure 8. Before the cantilever bracket was strengthened, the stress of the channel steel web near the baseboard was the highest, and the contact position between the channel steel and the baseboard was easy to be destroyed, as shown in Figure 8a. After the cantilever bracket was strengthened, the channel steel web with an additional square tube cantilever bracket and additional haunch had higher stress at the contact surface with the square tube or bracing, and there was obvious bending deformation, as shown in Figure 8b,c. The ultimate bearing capacity of the three models is 8481 N, 12,350 N, and 10,872 N, respectively.

**Figure 9:**
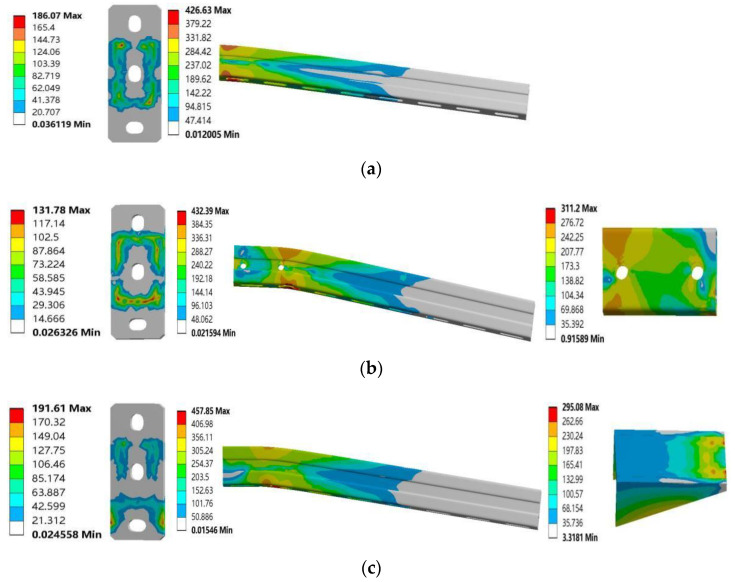
Stress cloud diagram of three kinds of cantilever brackets: (**a**) original cantilever bracket, (**b**) S1, (**c**) S2.

Figure 9 shows the stress distribution of the cantilever bracket after reaching the ultimate load. Figure 9a shows the stress distribution of each member of the ordinary cantilever bracket. Under the vertical load, the upper part of the channel steel was subjected to tension, and the lower part was compressed, the fixed end of the cantilever bracket received the largest bending moment, and the stress value of the channel steel web near the baseboard was 426.63 MPa. Figure 9b shows the stress distribution of each component of the additional square tube cantilever bracket, and the stress distribution in the channel steel tension zone and compression zone is relatively uniform. In the form of bolted fastening square pipe and channel steel, the channel steel was in close contact with the baseboard under vertical load, and the square pipe could bear a large part of the force, which plays a good role in the continuity of load transfer. The overall force of the cantilever bracket was reasonable, and the stress value of the contact part between the square tube and the channel web was 432.39 MPa. The stress distribution on the floor of the cantilever bracket with an additional square tube was more uniform; the reinforcement form of an additional square tube is beneficial to reduce the stress on the baseboard. Figure 9c shows the stress distribution of each component of the additional support cantilever bracket. The right-angle support provides upward force to the channel steel and plays a marvelous supporting role. The maximum stress at the edge of the contact edge between the right-angle support and the channel web can reach 457.85 MPa. It can be seen from the diagram that the stress values at the baseboard of the three types of cantilever brackets are all relatively small.

In contrast, by strengthening the cantilever bracket in the form of an additional square tube or additional haunch, the cantilever bracket can avoid damage in the position where the channel steel is in contact with the baseboard, the unsubstantial part of the cantilever bracket. The analysis of the numerical simulation results of the cantilever bracket shows that after adopting the reinforcement scheme, the stress distribution and stress mechanism of the cantilever bracket were better, and the reinforcement method based on optimizing the baseboard structure could improve the mechanical properties of the cantilever bracket.

## 4. Experiment and Discussion

### 4.1. Experimental Design

**Figure 10:**
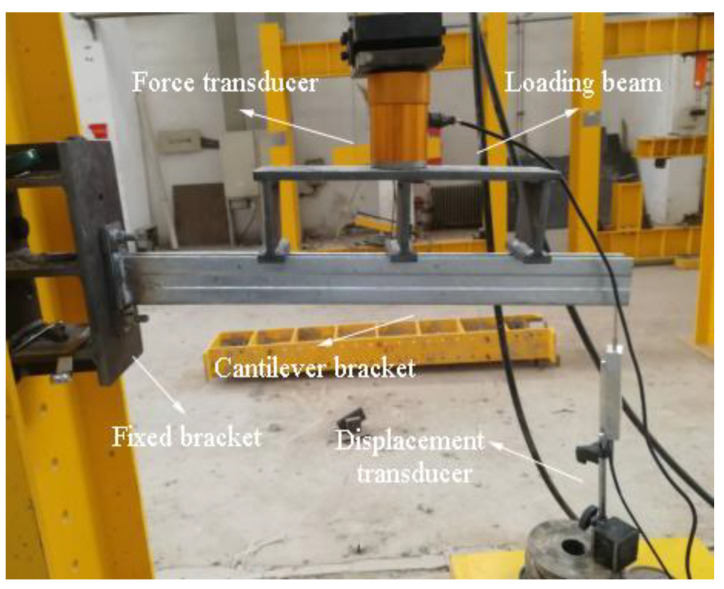
Schematic diagram of test loading.

This experiment was carried out in the structural laboratory of Henan University, and the quasi-static test was performed. The cantilever brackets were fixed to the ZF-FY30 reaction frame, and the hydraulic Jack exerted a load on the cantilever brackets by loading the beam. The span of the loading beam was 400 mm, as shown in Figure 10. The load–displacement data were collected by the force sensor and the displacement sensor, and the displacement sensor was fixed to the free end of the cantilever bracket. The test adopted the hierarchical loading system, and before the formal test loading, the debugged test system was preloaded to about 20% of the estimated ultimate bearing capacity to observe whether the test device and instrument operated normally. After ensuring that the test system was normal, the test was loaded formally with each stage load of 500 N, and the loading was continuous for 1 min and stops 3 min. The load–displacement data were collected, and the test phenomena were recorded. When the component was damaged, or the displacement was too large, the test finished.

### 4.2. Analysis of Experimental Phenomena and Results

**Figure 11:**
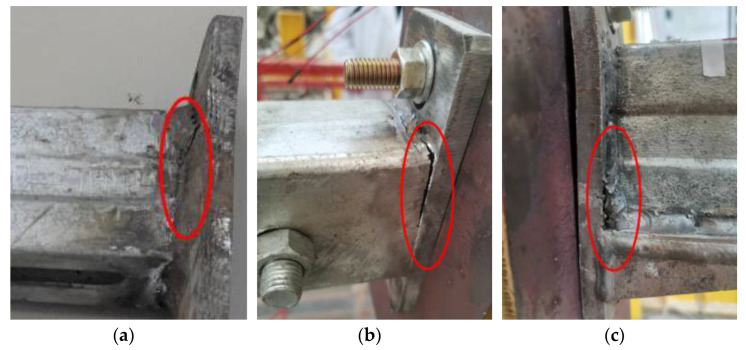
Details of failure components: (**a**) original cantilever bracket, (**b**) S1, (**c**) S2.

For the original cantilever bracket, when the load was about 4000 N, the baseboard was slightly deformed, and the channel steel free end slipped. When the load was 7000 N, the channel steel baseboard was slightly deformed, and the channel steel free end displacement reached 15.2 mm. When the load was further applied to 8100 N, the channel steel tilted, the free end displacement reached 75.3 mm, and there were small cracks in the weld between the channel steel and the baseboard. At this time, the connection between the Jack and the force sensor was not suitable for continuing loading, as shown in Figure 11a.

For the S1, the additional square tube cantilever bracket had no obvious test phenomenon at the initial stage of loading. When the load was applied to 5000 N, the square tube appeared to be slight deformation; when the load reached 9000 N, the square pipe slipped, and the free end displacement of channel steel was 14.4 mm. When the load continued to 11,200 N, the free end displacement of channel steel reached 62.3 mm, the bolt hole area was deformed, and the weld at the upper end of the square pipe was cracked, as shown in Figure 11b.

For the S2, the test phenomenon and failure situation of the bearing capacity of the cantilever bracket with additional support was similar to the former. When the load was about 4500 N, the baseboard had slight deformation, and the whole cantilever support had no obvious change. When the load value was 8500 N, the deformation of the channel steel baseboard increased, and the free end displacement of channel steel was 12.6 mm. When the channel steel was further loaded to 10,200 N, the free end displacement of the channel steel reached 59.5 mm, the baseboard bent obviously, and there were small cracks in the weld between the support, the channel steel, and the baseboard, as shown in Figure 11c.

**Figure 12:**
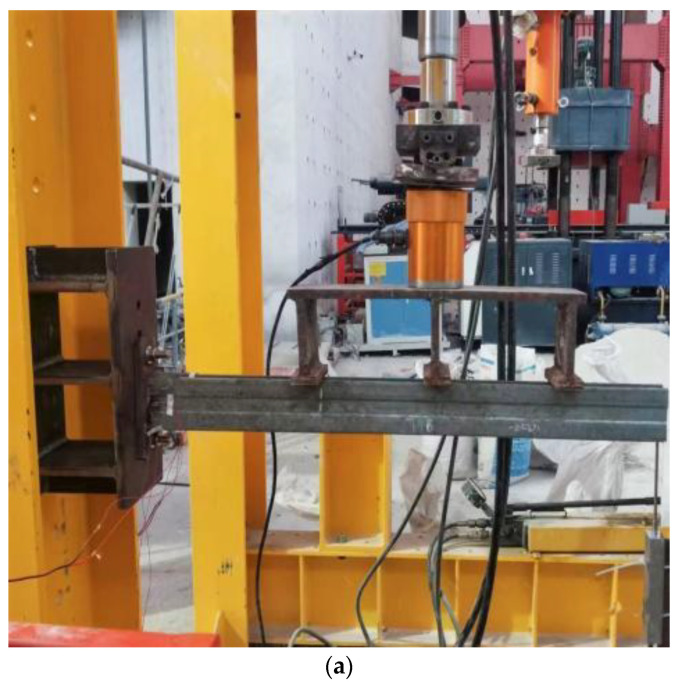

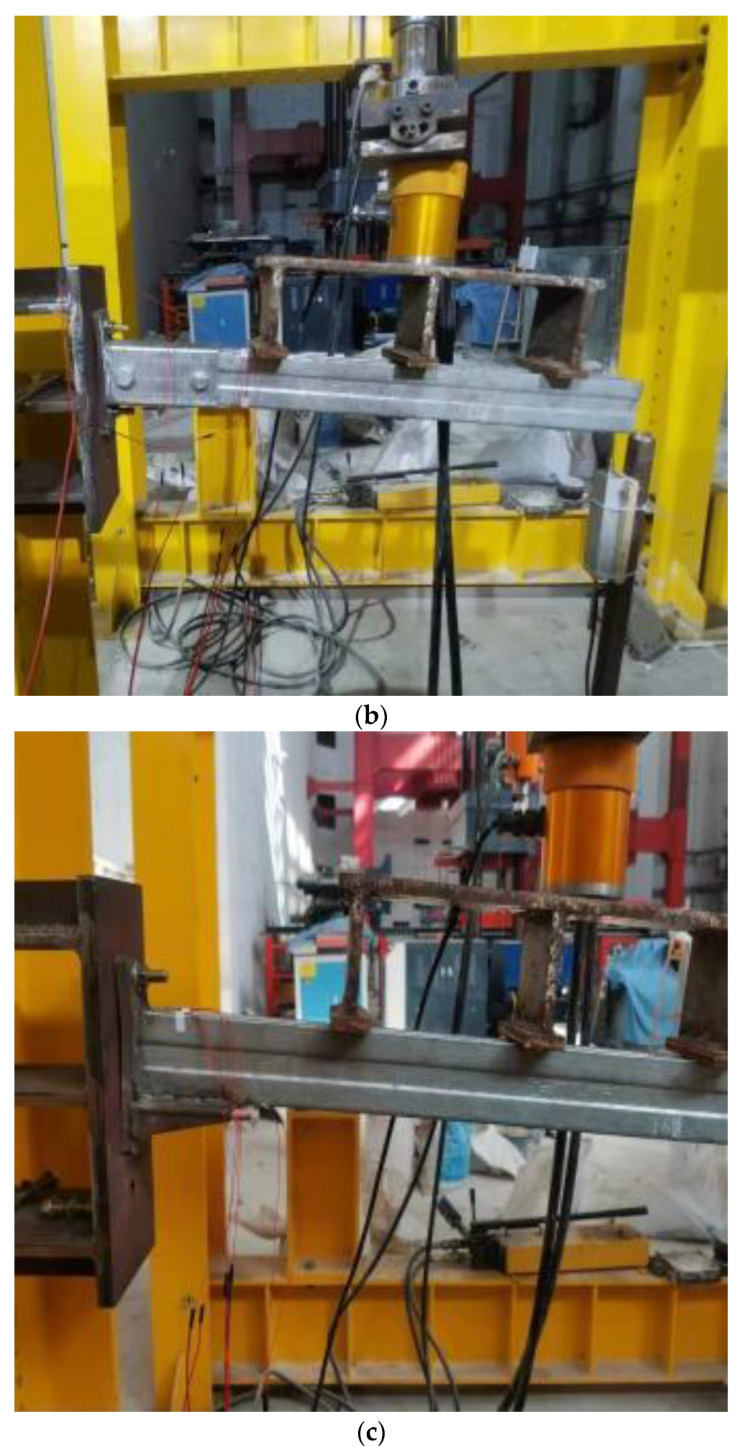
Loss of bearing capacity of test members: (**a**) original cantilever bracket, (**b**) S1, (**c**) S2.

The failure patterns of three kinds of cantilever brackets when they lose their bearing capacity are shown in Figure 12. When comparing the test results with the FE results, the failure modes are the approximation. During the test, the main reason for the loss of bearing capacity of the ordinary cantilever bracket was the cracking of the weld between the channel steel and the baseboard and the bending failure of the channel steel web near the baseboard. For the S1, under the initial load, the pressure on the channel steel was transferred to the square pipe hole wall through the bolt rod. With the increase in the load, the channel steel slipped downward, friction occurred between the channel steel wall and the square pipe wall, and the square pipe and the channel steel were in contact with each other bearing the load together. The main reason for the failure of the additional square tube cantilever bracket was the bending deformation at the edge of the contact between the channel steel and the square pipe and the cracking of the weld between the square tube and the baseboard. For the S2, when the member was destroyed, the bottom surface of the channel steel first yielded, the channel steel web and the contact edge of the support produced bending deformation, there was an obvious crease, and the baseboard appeared obvious bending deformation.

**Figure 13:**
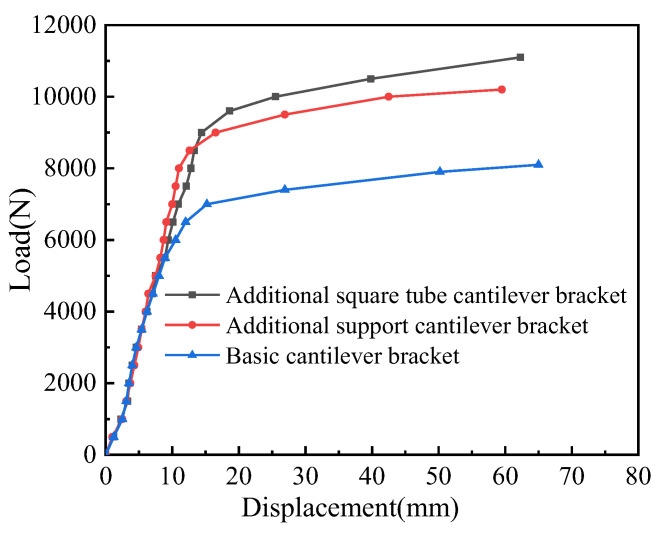
Test results of three types of cantilever bracket.

Figure 13 shows the load–displacement curve of cantilever brackets. It can be seen from the figure that the load–displacement curves of the three kinds of cantilever brackets almost coincide in the elastic stage. In contrast, the bearing capacity of the original cantilever bracket is the lowest, and the load–displacement curve first reaches the yield point; the structural stiffness decreases obviously after entering the yield stage. After entering the plastic strengthening stage, the free end displacement of channel steel increases rapidly, and the bearing capacity continues to increase. The bearing capacity of S1 is the largest. After entering the elastic-plastic stage, the structural stiffness of the cantilever bracket decreases, and when the member enters the plastic strengthening stage, the bearing capacity of the cantilever bracket increases. As a result, the reinforcement form of an additional square tube is beneficial to improving the bearing capacity and the structural stiffness of the cantilever bracket. The structural stiffness of S2 is the largest. After entering the elastic-plastic stage, the structural stiffness decreases slightly, and after entering the plastic strengthening stage, the bearing capacity of S2 increases.

In addition, from the load–displacement curve of the cantilever bracket, it can be seen that after entering the strengthening stage, the overall stiffness of S1 and S2 is higher than that of the original cantilever bracket, and the structural stiffness of S2 is the highest before reaching the yield load. However, after entering the strengthening stage, the structural stiffness of S1 is obviously larger. This shows that the reinforcement method of bolted square pipe is beneficial in slowing down the degradation of structural stiffness. The bolts and right-angle support in S1 and S2 are like the fulcrum of the lever, respectively, and the arm of the load applied by S1 is shorter. In the later stage of strengthening, if the load continues to be applied, the effect of S1 in increasing the ultimate bearing capacity will be more obvious, while the displacement of S2 increases faster.

After adopting the reinforcement scheme, the bearing capacity of the two new cantilever brackets was greatly improved, the yield load and ultimate load of S1 increased by 28.6% and 38.3%, respectively, and the yield load and ultimate load of S2 increased by 21.4% and 25.9% respectively. After entering the plastic strengthening stage, the bearing capacity increased steadily, which also shows that the ductility of the cantilever bracket was better after using the reinforcement scheme. The specific yield point, load, and displacement at the limit point of the three kinds of cantilever brackets are shown in Table 4.

### 4.3. Verification of Test Results and Finite Element Results

**Figure 14:**
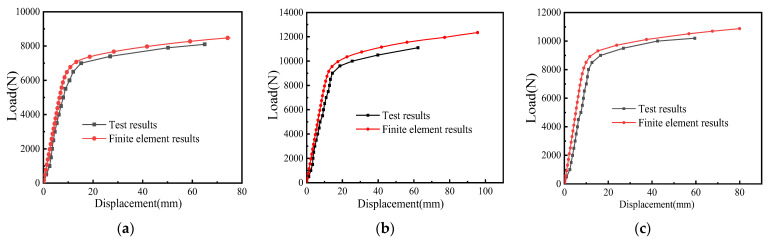
Comparison of load–displacement curves: (**a**) original cantilever bracket, (**b**) S1, (**c**) S2.

When comparing the FE results and test results of three kinds of cantilever brackets, we can see that the development trend of the load–displacement curve is consistent, and the two results fit well, as shown in Figure 14.

The FE simulation results of the original cantilever bracket in Figure 14a show that the member yielded when the load was applied to 7175 N, and the displacement value was 14.6 mm. After entering the strengthening stage, the load–displacement curve of the original cantilever bracket increased slowly, and when the load increased to 8481 N, the cantilever bracket lost its bearing capacity, and the displacement value was 75.3 mm. From the load–displacement curve of the test results, it can be seen that there is an obvious yield point when the load reaches 7000 N, and then the increase in bearing capacity tends to be gentle. When the load continues to load to 8200 N, the displacement is 65 mm. Due to the large displacement of the free end of the cantilever bracket, the channel steel is obviously inclined, and the test system is no longer suitable for loading. When comparing the FE results with the test results, the error was 2.5%.

In Figure 14b, the FE model of S1 appears to have an obvious inflection point at 9350 N, and the displacement is 13.1 mm when the member changes from the yield stage to the strengthening stage. When the load continues to 12,350 N, the member completely loses its bearing capacity. From the test results, it can be seen that there is obvious yield when the load is 9000 N, and after entering the strengthening stage, the displacement of the bracket increases sharply, showing obvious plasticity and stiffness decreases obviously. When the load reaches 11,200 N, the displacement reaches 62.3 mm, and the test system is no longer suitable for loading. The error of the yield load between the FE analysis results and the test results is 3.9%.

In Figure 14c, S2 yielded when the load was applied to 8912 N, and the displacement value was 11.6 mm. After entering the strengthening stage, the load–displacement curve of the additional support cantilever bracket increased slowly. When the load increased to 10,872 N, the bracket lost its bearing capacity. From the load–displacement curve of the test results, it can be seen that there is an obvious yield point when the load reaches 8500 N, and then the increase in bearing capacity tends to be gentle. When the load continues to load to 9800 N, the displacement value is 59.5 mm. The displacement of the cantilever brackets is large, the channel steel is obviously inclined, and the test system is no longer suitable for loading. The error of the yield load between the FE results and the test results is 4.8%.

When comparing the test results and FE simulation results of the three kinds of cantilever brackets, it can be seen that the ultimate bearing capacity of the bracket with a square tube is the largest, and the structural stiffness is slightly lower because the bolt is not a completely rigid connection. The channel steel produces a microrotation under the load, and the friction between the channel steel and the square tube also consumes part of the internal force, thus improving the bearing capacity of the bracket. The structure of S2 is simple; the gap between the members is less, the stiffness of the whole structure is the largest, and the initial displacement is small.

There are some errors between the test results and the FE results for three main reasons. Firstly, there are gaps in the assembly of the assembled cantilever brackets; for example, because the support baseboard uses an oval bolt hole, the bolt produces certain gaps in the installation process. Secondly, the model itself also has some defects, so under the initial load, the initial displacement value of the test is larger than the FE simulation value. Thirdly, due to the limitations of the test loading device, once the angle between the Jack and the force sensor interface appears, the test system cannot continue to load. Through the analysis of the load–displacement curves of three kinds of cantilever brackets, it is known that after entering the strengthening stage, the development trend of the test results is consistent with the FE simulation. In summary, the yield-bearing capacity, failure mode, and deformation process of the test results are in good agreement with the FE simulation, which verifies the correctness of the FE model and can be used as the benchmark FE model.

## 5. Conclusions

When more communication cables are laid on the existing supports, it is necessary to take measures for the original cantilever bracket. The two reinforcement schemes provided in this paper are of great help to improving the bearing capacity of the cantilever bracket and enhancing the structural stiffness of the members:

(1) Comparatively speaking, the bearing capacity of S1 is the largest, and the yield load and ultimate load are increased by 28.6% and 38.3%, respectively, compared with the foundation cantilever bracket, while the structural stiffness of S2 is the largest;

(2) Through the study, it can be concluded that the thickness of the baseboard has an effect on the bearing capacity of the original cantilever bracket and S2, but there is no obvious bending deformation on the baseboard of S1. As a result, it can be concluded that the fastening form of the additional square tube can reduce the force on the baseboard of the cantilever bracket and appropriately reduce the thickness of the baseboard of the S1, which can also meet the design requirements of bearing capacity;

(3) The results show that S1 and S2 are still steady after entering the strengthening stage, indicating that the cantilever bracket still has good ductility after strengthening measures;

(4) The load–displacement curves of the three kinds of cantilever brackets fit well, and the model verified by the test results can be used as the basic finite element model to study the other mechanical properties of the cantilever bracket.

## Figures and Tables

**Table 1:** Dimensions of components composed of two types of cantilever bracket.

Component Name	Channel Steel (mm)	Baseboard (mm)	Square Sleeve Pipe (mm)	Transverse Plate (mm)	Right-Angle Plate (mm)
S1	72 × 42.3 × 2.75	165 × 60 × 12	150 × 50 × 80	-	-
S2	72 × 42.3 × 2.75	165 × 60 × 12	-	100 × 60 × 6	100 × 31 × 6

**Table 2:** Mechanical properties parameters of materials.

Component Name	Density (t/mm^3^)	Elastic Modulus (GPa)	ν	$\sigma_{s}$ (MPa)	E_T_ (GPa)
Channel steel	7.80 × 10^−9^	210	0.3	270	0.01E
Baseboard/Square tube/Support	7.80 × 10^−9^	206	0.3	235	0.01E
M16 Bolt	7.80 × 10^−9^	206	0.3	640	0.01E

**Table 3:** Error analysis of FE results.

Nodes of Mesh	Py (N)	Error	$\Delta_{y}$ (mm)	Error	P_u_ (N)	Error	$\Delta_{u}$ (mm)	Error
119,300	9350	-	13.10	-	12,350	-	95.626	-
133,243	9350	0%	12.96	1.1%	12,350	0%	94.804	0.9%
181,165	9550	2.1%	13.76	5%	12,350	0%	97.15	1.5%

**Table 4:** Test results of components.

Test Component	P_y_ (N)	$\Delta_{y}$ (mm)	P_u_ (N)	$\Delta_{u}$ (mm)
Ordinary cantilever bracket	7000	15.2	8100	65
Additional square tube cantilever bracket	9000	14.4	11,200	62.3
Additional support cantilever bracket	8500	12.6	10,200	59.5

## Data Availability

Some or all data, models, or codes that support the findings of this study are available from the corresponding author upon reasonable request.
